# SIP1 is downregulated in hepatocellular carcinoma by promoter hypermethylation

**DOI:** 10.1186/1471-2407-11-223

**Published:** 2011-06-06

**Authors:** Tolga Acun, Emin Oztas, Tamer Yagci, Mustafa C Yakicier

**Affiliations:** 1 Bilkent University, Department of Molecular Biology and Genetics, 06800 Ankara, Turkey; 2 Department of Histology and Embryology, Gulhane Military Medical Academy (GMMA), 06018 Ankara, Turkey; 3 Gebze Institute of Technology, Department of Molecular Biology and Genetics, 41400 Cayirova, Kocaeli, Turkey; 4 Department of Medical Biology, Acibadem University, 34848 Istanbul, Turkey

## Abstract

**Background:**

Smad interacting protein-1 is a transcription factor that is implicated in transforming growth factor-β/bone morphogenetic protein signaling and a repressor of E-cadherin and human telomerase reverse transcriptase. It is also involved in epithelial-mesenchymal transition and tumorigenesis. However, genetic and epigenetic alterations of *SIP1 *have not been fully elucidated in cancers. In this study, we investigated mutations and promoter hypermethylation of the *SIP1 *gene in human hepatocellular carcinomas.

**Methods:**

*SIP1 *expression was analyzed in HCC cell lines and primary tumors in comparison to normal and non-tumor liver tissues by using semi-quantitative RT-PCR, quantitative real-time RT-PCR and immunohistochemistry. Mutation and deletion screening of the *SIP1 *gene were performed by direct sequencing in HCC-derived cells. Restoration of *SIP1 *expression was sought by treating HCC cell lines with the DNA methyl transferase inhibitor, 5-AzaC, and the histone deacetylase inhibitor, TSA. *SIP1 *promoter methylation was analyzed by the combined bisulfite restriction analysis assay in *in silico*-predicted putative promoter and CpG island regions.

**Results:**

We found that the expression of *SIP1 *was completely lost or reduced in five of 14 (36%) HCC cell lines and 17 of 23 (74%) primary HCC tumors. Immunohistochemical analysis confirmed that *SIP1 *mRNA downregulation was associated with decreased expression of the SIP1 protein in HCC tissues (82.8%). No somatic mutation was observed in *SIP1 *exons in any of the 14 HCC cell lines. Combined treatment with DNA methyl transferase and histone deacetylase inhibitors synergistically restored *SIP1 *expression in *SIP1*-negative cell lines. Analysis of three putative gene regulatory regions revealed tumor-specific methylation in more than half of the HCC cases.

**Conclusions:**

Epigenetic mechanisms contribute significantly to the downregulation of *SIP1 *expression in HCC. This finding adds a new level of complexity to the role of SIP1 in hepatocarcinogenesis.

## Background

Hepatocellular carcinoma (HCC) is one of the most lethal cancer types worldwide and also the most common type of liver cancer [1-3]. The exact mechanisms that drive hepatocarcinogenic processes are not yet completely understood. Identification of genetic and epigenetic changes involved in hepatocellular carcinoma development is of high interest for a better understanding of this aggressive malignancy.

Smad interacting protein-1 (SIP1, also known as ZEB2) is encoded by *ZFHX1B *at chromosome 2q22 and is a two-handed zinc finger transcription factor that contains a central homeodomain as well as CtBP-binding and Smad-interacting domains. SIP1 has been shown to act predominantly as transcriptional repressor but can also act as transcriptional activator *in vivo *[4-8].

SIP1 was originally identified in a transforming growth factor-β/bone morphogenetic protein (TGF-β/BMP) signaling pathway by its binding to the MH2 domain of receptor-activated SMADs [9]. SIP1 has been thoroughly studied for its role in repressing E-cadherin expression, which is a central event in the epithelial-to-mesenchymal transition (EMT) [5-7,10,11]. Accordingly, an elevated SIP1/E-cadherin ratio was shown to correlate with invasive disease and poor prognosis in gastric, pancreatic, esophageal and ovarian carcinomas [12-15]. Overexpressed *SIP1 *also caused resistance to DNA damage-induced apoptosis and correlated with poor survival in patients with bladder cancer [16]. In contrast, only a few studies exist with regard to the role of SIP1 in suppressing tumorigenesis. For instance, repression of human telomerase reverse transcriptase (*hTERT*) expression in breast and liver cancer cells was shown to be partly mediated by SIP1 [17,18]. Also, by directly inhibiting cyclin D1, SIP1 caused G1 arrest in squamous carcinoma cells [19].

*SIP1 *was strongly expressed in, and with another transcriptional repressor, *SNAIL*, increased invasion of HCC cells [20]. We recently reported an immunohistochemistry study on tissue arrays and described decreased SIP1 levels in a group of tumors, including HCC [21]. In mature hepatocytes *in vitro*, TGF-β induces EMT by downregulation of Claudin-1, which is also associated with upregulation of *SIP1 *and *SNAIL *and downregulation of E-cadherin [22]. Our recent observations also implicated *SIP1 *as a candidate regulator of replicative senescence in HCC cells [18]. Taken together, these findings indicate that *SIP1 *may play a role in hepatocarcinogenesis.

Epigenetic regulation of *SIP1 *expression by miRNAs [23-26] and a natural antisense transcript (NAT) [27] were recently described. Studies on the promoter methylation of *SIP1 *were also reported. The *SIP1 *gene was found to be hypermethylated and silenced in a poorly metastatic breast cancer cell line [28]. In a more recent study, *SIP1 *downregulation in pancreatic cancer was shown to be mediated through promoter hypermethylation [29]. However, genetic and epigenetic mechanisms regulating *SIP1 *expression have never been studied in HCC.

In the present study, we investigated the expression of *SIP1 *at genetic, epigenetic and protein levels in a series of HCC cell lines and primary tumors. Downregulation of *SIP1 *in HCC cell lines and tumors was found to be mediated by aberrant promoter methylation. Therefore, epigenetic inactivation of *SIP1 *may play a critical role in hepatocarcinogenesis.

## Methods

### Cell lines and patient samples

DNA samples from 39 pairs of HCCs and tumor-adjacent normal tissues were used; these archival materials have previously been described [30]. HCC-derived SNU449, SNU475, Mahlavu, SNU423, SNU398, SK-Hep1, Focus, SNU387, SNU182, Hep40, Huh7, PLC/PRF5, Hep3B and hepatoblastoma-derived HepG2 cell lines were studied.

TissueScan Liver Cancer Tissue qPCR Panel I was purchased from Origene Technologies (Rockville, MD). Each plate consisted of pre-normalized cDNAs derived from 48 liver samples covering eight tumor-adjacent normal, 23 HCC (grade I, II, IIIA, IV), three cholangiocarcinoma, one adenoma and 13 non-tumor lesions of the liver. (Additional file 1).

Fifteen formalin-fixed and paraffin-embedded liver tissues deriving from two normal, one HBV carrier, three chronic hepatitis B, three chronic hepatitis C, three cirrhosis and three HCC cases were obtained from the Department of Pathology of Gulhane Military Medical Academy in Ankara, Turkey, upon the favourable decision of ethics committee of Gulhane Military Medical Academy. An HCC tissue array consisting of 61 tumors and five normal cases was purchased from Biochain (Hayward, CA). A total cell lysate of a normal liver tissue was kindly provided by Dr KC Akcali.

### Multiplex semi-quantitative RT-PCR

Total RNA was isolated from cell lines using NucleoSpin RNA II (Macherey-Nagel, Germany) as indicated by the manufacturer. Reverse transcription was performed with Revert Aid First Strand cDNA Synthesis kit (Fermentas, Lithuania) according to the manufacturer's instructions. Multiplex semi-quantitative RT-PCR was performed by using *SIP1*-specific primers (SIP1-RTF and SIP1-RTR) and *GAPDH*-specific primers (GAPDH-RTF and GAPDH-RTR). Primer sequences are given in the supplementary information section (Additional file 2).

### Quantitative RT-PCR

Quantitative analysis of *SIP1 *transcripts was performed by real-time RT-PCR on cDNAs from the TissueScan liver cancer tissue qPCR panel I (OriGene, Rockville, MD) according to the manufacturer's instructions and as previously described [31]. Finnzymes DyNAmo™ HS SYBR Green qPCR kit (Finnzymes, Finland) was used and samples were run on a Stratagene MX3005P™ real-time PCR system (Stratagene, La Jolla, CA). The *TBP *gene was used as an internal control [32,33] and relative levels of *SIP1 *transcripts were measured by a modified ΔΔCt formula [31,34]. Statistical analyses were performed by the Student's t test.

### Western blotting

Western blotting was performed as previously described [21]. Briefly, total cell lysates from SNU398, Huh7, Hep40 and SNU182 cell lines were prepared in NP-40 lysis buffer [50 mM Tris-HCl pH 8.0, 150 mM NaCl, 1% Non-idet P40 (v/v) and a cocktail of EDTA-free protease inhibitors (Roche Diagnostics, Mannheim, Germany)]. Protein content was measured with the Bradford assay. Equalized lysates were run on 8% SDS-PAGE and then transferred onto polyvinylidene fluoride (PVDF) membranes using a wet transfer apparatus (Bio-Rad, Hercules, CA). 6E5 hybridoma supernatant was used as the primary antibody. Horseradish peroxidase (HRP)-conjugated donkey anti-mouse IgG (sc-2318; Santa Cruz, CA) was used as the secondary antibody at 1:2000 dilution. Protein bands were visualized using SuperSignal West Femto chemiluminescent substrate (Pierce, Rockford, IL).

### Immunohistochemistry

Following a pathologist's review, immunohistochemistry (IHC) was performed on human liver tissues to determine the expression of SIP1, as previously described [21]. In brief, tissue sections were deparaffinized and treated with 10 mM citrate buffer for antigen retrieval. Then, samples were incubated first with a homemade SIP1 monoclonal antibody (clone 6E5), and after washing, with a universal staining kit secondary reagent (LabVision, Fremont, CA). An IgG2a isotype antibody was used as the control (R&D Systems, MN). Diaminobenzidine (DAB) was used as the chromogen, and the slides were counterstained using Mayer's hematoxylin. Immunoreactivity was registered semi-quantitatively, and the immunostaining in each section was assessed independently by two observers (EO and TY). The staining intensity was graded relatively as: no staining (0), weak (+), moderate (++) or strong (+++) [35].

### Mutation screening

Except UTR, all exons of *SIP1 *were amplified in 14 HCC cell lines using 13 sets of primers (Additional file 2). PCR products were purified with the PCR_96 _Cleanup Kit (Millipore, Billerica, MA) and directly sequenced by the sequencing service company Iontek (Istanbul, Turkey). Mutation screening was performed using the Mutation Surveyor software package (v 3.10, SoftGenetics, LLC, State College, PA).

### 5-azacytidine and trichostatin A treatment of HCC cell lines

5-azacytidine (5-AzaC) and Trichostatin A (TSA) treatments were performed as described previously [36-38]. Briefly, cells were seeded in six-well plates at a density of 3 × 10^5 ^cells/well and treated after 24 h with 2.5 μM 5-AzaC (Sigma-Aldrich, St. Louis, MO) for 96 h. In the last 24 h, they were treated with 300 nM or 1 μM TSA (Sigma-Aldrich, St. Louis, MO) either alone or combined with 5-AzaC. The medium and 5-AzaC were refreshed every 24 h. Control cultures were left untreated or received a mock treatment with adequate volumes of DMSO (Sigma-Aldrich, St. Louis, MO) in the case of TSA treatment. At the end of the treatment, cells were harvested for DNA and RNA isolation.

### CpG island search in SIP1 promoter

The sequence of the human *SIP1 *gene was retrieved from the NCBI gene database and putative *SIP1 *promoters were predicted by Promoter2.0 software [39]. The genomic region containing the *SIP1 *gene, starting from the third exon to 20 kb upstream of the first exon, was analyzed for CpG islands by MethPrimer software [40].

### Sodium bisulfite treatment and combined bisulfite restriction analysis (COBRA)

Genomic DNA was extracted from the cell lines using the Qiagen DNeasy Tissue kit (Hilden, Germany) and bisulfite treated with the Epigentek Methylamp™ DNA Modification kit (Brooklyn, NY) according to the manufacturers' instructions. Nested primer pairs targeted to *SIP1 *CpG islands were used to amplify bisulfite-treated DNAs (the list of primers is given in Additional file 2) and PCR products were restriction digested by *Bst*UI or *Taq*I (New England BioLabs, Ipswich, MA) to detect methylation status, as previously described [41].

## Results

### Differential *SIP1 *expression in HCC cell lines

**Figure 1:**
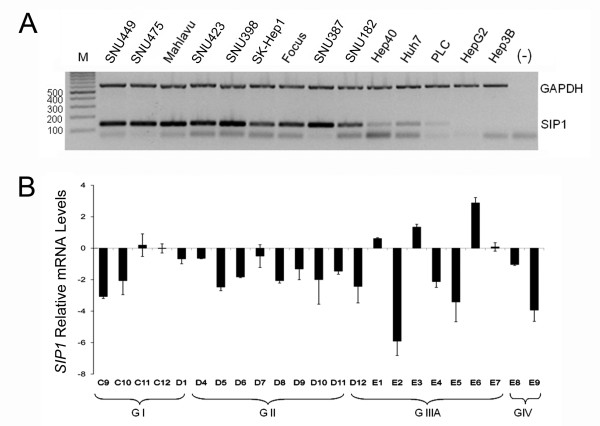
**Expression of *SIP1 *in HCC cell lines and tumors**. **(A) ***SIP1 *expression is detected in 14 HCC cell lines by multiplex semi-quantitative RT-PCR. GAPDH is used as an internal control. Amplicons corresponding to *SIP1 *and *GAPDH *are 132 bp and 611 bp, respectively. A negative control without a DNA template is included. Bands lower than 100 bp in most of the cell lines and negative control sample are primer dimers. M: marker. **(B) ***SIP1 *mRNA expression in HCC tumors relative to tumor-adjacent and normal tissues is analyzed by quantitative RT-PCR. Relative mRNA levels of *SIP1 *normalized to TBP are represented. Patients are grouped depending on tumor stage (GI-GIV). Clinicopathological characteristics of patients (C9-E9) are given in Additional file 1. Standard deviations of two independent experiments are shown.

We first identified the mRNA expression of *SIP1 *in 14 HCC cell lines by multiplex semi-quantitative RT-PCR (Figure 1A). In our experimental settings, the *SIP1 *transcript was absent in the Hep3B and HepG2, very low in the PLC/PRF/5 and weak in the Hep40 and Huh7 cell lines. The other nine cell lines, SNU449, SNU475, Mahlavu, SNU423, SNU398, SK-Hep1, Focus, SNU387 and SNU182, displayed much stronger *SIP1 *mRNA expression. These results suggested a negative regulation of *SIP1 *in some HCC cells by as yet unidentified mechanisms.

### *SIP1 *expression is reduced in human HCCs

We expanded our analysis to clinical samples in order to detect the expression of *SIP1 *transcripts in a panel of human HCCs by quantitative real-time RT-PCR (Figure 1B). *SIP1 *expression was found to be significantly reduced in 17 of 23 (73.91%) HCC tumors, compared to eight normal liver tissues from the same panel (p = 0.01). Three of the remaining HCC samples displayed normal and the other three showed high *SIP1 *transcript levels. Decreased *SIP1 *expression was also observed in the majority of the non-HCC liver lesions - the cholangiocarcinomas and adenomas (data not shown).

### SIP1 protein is missed or downregulated in HCCs

**Figure 2:**
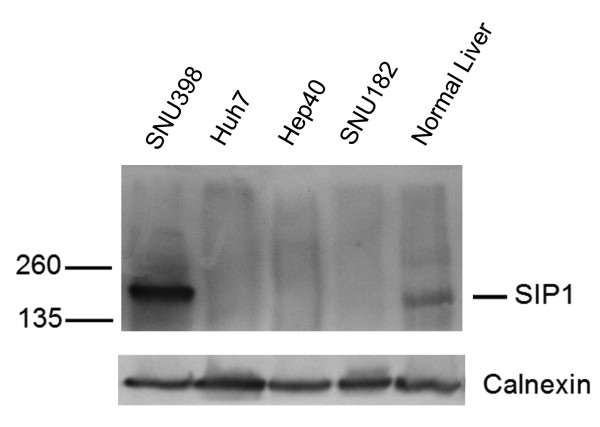
**Endogenous expression of SIP1 protein in HCC cell lines and normal liver**. The specificity of 6E5 monoclonal antibody is assessed by a Western blot experiment by using total cell lysates from HCC cell lines (SNU398, Huh7, Hep40, SNU182) and a normal liver. An SIP1 protein band at the expected size is observed only in SNU398 and normal liver cells.

**Figure 3:**
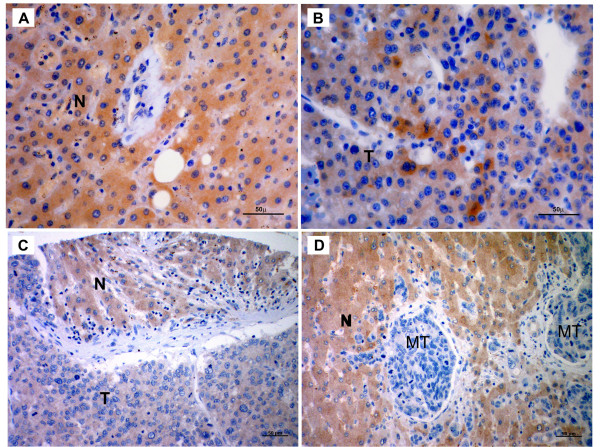
**SIP1 protein expression in human liver tissues**. Photographs are representative of SIP1 immunohistochemistry in human liver tissues. (**A**) Normal tissue shows strong, diffuse cytoplasmic staining. (**B**) Heterogenous expression of SIP1 in HCC. Tumor section displays faint immunostaining, except for a few cells with strong immunoreactivity. (**C**) A tissue array sample includes both tumor and non-tumor cells that differentially express SIP1. (**D**) Adenocarcinoma metastasis to liver. While metastatic cells are negatively stained, hepatocytes strongly express SIP1. N: Normal hepatocytes; T: HCC cells; MT: Metastatic adenocarcinoma cells (Scale Bars: 50 μm).

A previously described, an anti-SIP1 monoclonal antibody, clone 6E5, was used in the IHC experiments [21]. The specificity of the antibody was first assessed by Western blotting in HCC cell lines and a control liver tissue (Figure 2). Except for cells from the poorly differentiated SNU398 cell line and the normal liver, none of the HCC cells displayed SIP1 protein expression. Next, IHC was performed on liver tissue array and archival human liver tissue sections, including non-HCC (normal liver, chronic hepatitis, cirrhosis) and HCC samples (Figure 3). We observed decreased expression of SIP1 in HCC cases compared to non-HCC tissues. Hepatocytes from all 17 non-HCC tissues showed moderate and strong SIP1 cytoplasmic expression. In sharp contrast, no SIP1 protein expression was found in 53 of 64 (82.8%) HCC cases. In addition, tumors in the remaining 11 cases (17.2%) displayed only weak immunoreactivity. The IHC results of the liver tissues are summarized in Table 1.

**Table 1:** Immunostaining of human liver tissues with SIP1 antibody.

	SIP1 Expression (staining intensity)		
	
Pathological Diagnosis	No staining	(+)	(++/+++)
HCC (n = 64)	53 (82.8%)	11 (17.2%)	
Normal Liver (n = 7)			7 (100%)
Cirrhosis (n = 3)			3 (100%)
HBV Carrier (n = 1)			1 (100%)
Chronic Hepatitis			
HBV (n = 3)			3(100%)
HCV (n = 3)			3 (100%)

### Absence of *SIP1 *mutations in HCCs

To investigate whether *SIP1 *is inactivated by allelic deletions and/or somatic mutations, we performed direct sequence analysis using genomic DNA from 14 HCC cell lines. Genomic PCR was carried out using 13 sets of primers that amplify the entire coding region, including splice acceptor and donor sites (exons 2-10) of *SIP1*. No somatic mutations leading to amino acid substitutions or frameshifts were found, indicating that mutational alterations of *SIP1 *are not main genetic events in hepatocarcinogenesis (**data not shown**).

### Restoration of *SIP1 *mRNA expression by 5-AzaC and TSA treatments

**Figure 4:**
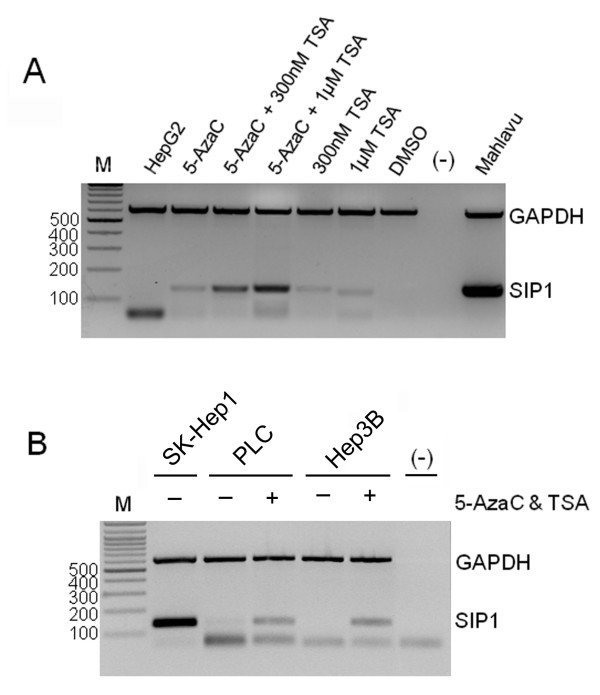
**Treatment with 5-AzaC and TSA rescues *SIP1 *expression in HCC cell lines**. The restoration of *SIP1 *expression is analyzed by multiplex semi-quantitative RT-PCR. **(A) **Treatment of HepG2 cells with 5-AzaC (2.5 μM) and TSA. Mahlavu cells are included as a positive control of SIP1 expression. DMSO is used as a control of TSA treatment and a DNA-free PCR mixture [(-)] is also tested. **(B) **PLC and Hep3B cell lines are treated with 2.5 μM 5-AzaC and 1 μM TSA. The SkHep1 cell line was used as a positive control. [(-)]: DNA-free PCR mixture. M: marker (bp). In both (A) and (B), bands lower than 100 bp are primer dimers.

The absence of *SIP1 *mutations in HCC cell lines prompted us to explore the role of DNA methylation and/or histone deacetylation as mechanisms that operate on *SIP1 *downregulation in HCC. To this end, we examined rescued *SIP1 *expression in three cell lines with decreased *SIP1 *transcripts upon treatment with DNA methyltransferase inhibitor 5-AzaC and histone deacetylase inhibitor TSA, either alone or in combination. When treated with 5-AzaC and TSA alone, *SIP1 *expression was found to be restored in the HepG2 cell line, and combined treatment substantially increased *SIP1 *transcript compared to cells treated with either agent alone (Figure 4A). Similar treatment conditions also restored the expression of *SIP1 *in Hep3B and PLC cells (Figure 4B).

### Frequent methylation of *SIP1 *promoter in primary HCC samples

**Figure 5:**
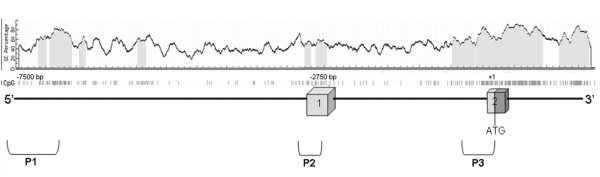
***In silico *analysis of CpG-rich putative *SIP1 *promoters**. The 100 Kb genomic region upstream of the third exon is analyzed by CpG island prediction (MethPrimer) and promoter prediction (Promoter2.0) programs. Localization of the CpG islands (gray zones) and three putative promoter (P1-P3) regions are represented. Boxes indicate the first and second exons and the translation start site of *SIP1*.

The restoration of *SIP1 *expression with the demethylating agent 5-AzaC is an indicator of promoter hypermethylation; we next aimed at analyzing the methylation status of the *SIP1 *promoter region. First, we sought out CpG islands by an *in silico *search in a ~100 kb region, from the third exon to 20 kb upstream of the first exon of *SIP1*, by using MethPrimer software [40]. Found CpG islands that accumulate in three distinct zones of the analyzed 5' site of the *SIP1 *gene are represented in Figure 5. Analysis of the same region by a promoter prediction program, Promoter 2.0 [39], revealed three previously described promoter candidates [42,43]. We noticed that putative promoters overlapped with some of the aforementioned CpG islands and we restricted our methylation analyses to these three gene regulatory regions (Figure 5).

**Figure 6:**
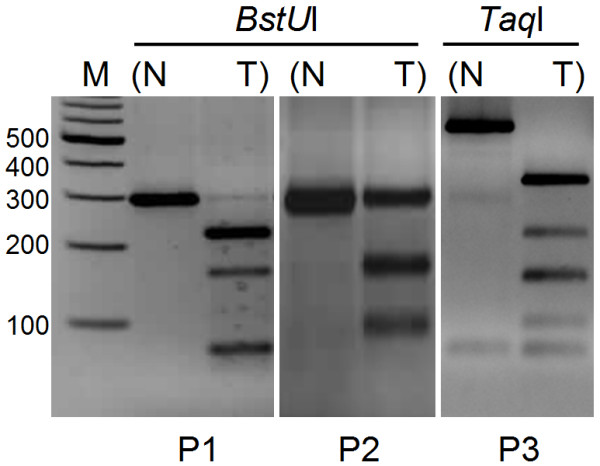
**Methylation analysis of promoter regions by COBRA**. Photographs are representative of tumor-specific methylation in three promoter regions. Amplicons of P1 and P2 are cut with *Bst*UI (left and middle), and *Taq*I digestion is applied to the PCR products of the P3 region (right). N: normal; T: tumor; M: marker.

To determine the overall frequency of *SIP1 *methylation in clinical HCC samples, we examined 39 tumor and paired normal liver tissues and performed COBRA analysis whenever an amplicon was obtained. Depending on the availability of restriction sites, PCR products of P1 and P2 were digested by *Bst*UI, and amplicons deriving from P3 were cut by *Taq*I enzymes (Additional file 3). A tumor-specific methylation pattern was observed in 48% (14/29) and 43% (10/23) of P1 and P3 sites, respectively. However, in our analysis of the P2 region, only 4% (1/26) of HCC samples were hypermethylated (Figure 6). COBRA analysis of the P1 and P3 regions also revealed partial methylation in both normal and tumor tissues in 52% (15/29) and 30% (7/23) of the paired samples, respectively. Six out of 23 amplicons in the P3 region (26%) failed to be restriction digested. We also noticed that 65.6% (21/32) of the paired samples displayed tumor-specific methylation when all three regions were considered. COBRA results of all paired HCC samples are given in Additional file 4.

## Discussion

SIP1 is a member of the ZEB family of transcription factors and, along with other E-cadherin repressors, it was repeatedly shown to induce the EMT phenotype both *in vivo *and *in vitro *and correlate with a poor prognosis in cancer patients [5,12,15,19]. On the other hand, SIP1 was also shown to be a negative regulator of *hTERT *transcription in breast cancer cells [17]. Consistent with this, we have recently reported that SIP1 was partly responsible for inducing senescence in hepatocellular carcinoma-derived cells through *hTERT *repression, and hypothesized that it may act as a tumor suppressor gene in HCC [18]. In support of this hypothesis, a recent microarray study showed downregulation of *SIP1 *in early and advanced HCC [44]. Also, induced expression of SIP1 has recently been shown to directly inhibit cyclin D1 in the A431 squamous carcinoma cell line, leading to the accumulation of cells in the G1 phase [19]. Other studies described posttranscriptional regulation mechanisms, such as those mediated by miR-200 family [24-26] and *SIP1 *NAT [27], in the downregulation of SIP1 in different pathophysological contexts.

Obviously, DNA methylation and alterations of chromatin structure are predominant mechanisms that epigenetically inactivate tumor suppressor genes in tumors [45]. For instance, *SIP1 *was found to be hypermethylated in poorly metastatic breast adenocarcinoma cells, but hypomethylated in a more aggressive variant of this cell line [28]. Yet more recently, silencing of *SIP1 *expression was shown to be mediated by promoter hypermethylation in a substantial proportion of pancreatic cancer cell lines and tissues [29].

Herein, we explored the expression of SIP1 in HCC at the transcriptional and protein levels and provided a mechanistic insight by demonstrating that promoter hypermethylation operates as one of the mechanisms in the epigenetic regulation and downregulation of SIP1 in the majority of HCC samples.

Our initial expression studies in HCC cell lines revealed two groups of cells that differentially express *SIP1*. Most fibroblastoid-like cells displayed strong *SIP1 *transcripts, while cell lines with an epitheloid appearance had no or low expression. This *in vitro *expression pattern of *SIP1 *was in accordance with its role in inducing EMT, but was neither informative about a tumor versus normal comparison of SIP1 levels nor the behaviour of SIP1 in a liver tissue context. We therefore proceeded with normal liver and HCC tissues and found a significant decrease of *SIP1 *transcripts in 74% of tumors. By using a previously described anti-SIP1 monoclonal antibody, clone 6E5, an immunoblot was performed with the lysates of HCC cell lines [21]. This assay not only proved the specificity of this antibody, but confirmed our initial observation that SIP1 is downregulated in tumors (Figure 2). Even a higher rate of *SIP1 *downregulation was observed in our IHC experiments. Compared to normal, 83% of HCC cases displayed no immunoreactivity and the remaining tumor samples were stained with only a weak intensity. This small difference between transcript and protein levels might be explained by the aforementioned posttranscriptional regulatory mechanisms of *SIP1 *expression. In fact, a miRNA profiling study that showed upregulation of miR-200c in HCCs but not in benign liver tumors could partly explain the downregulation of SIP1 in HCCs [46]. It would be interesting to analyze the expression levels of these regulators in HCCs.

Genetic screening of cell lines did not reveal any mutational alterations in the *SIP1 *gene, suggesting the implication of epigenetic regulatory mechanisms for the silencing of *SIP1 *in HCC. Upon our observation that *SIP1 *mRNA expression was restored after treatment of cells with 5-azaC and TSA, we decided to explore promoter hypermethylation as a possible mechanism of *SIP1 *downregulation in HCC.

*In vitro *activities of three alternative *SIP1 *promoter regions have been previously described in experiments with mouse tissue and the one located around the first exon (P2) exhibited the highest activity. The other alternative promoter regions (P1 and P3) were of low, but detectable, activity [42]. Studies in human cancer cell lines revealed a similar pattern of promoter activation, but this time in an AKT-dependent manner [43]. In striking contrast to these findings, we detected only one methylated tumor sample among 26 paired tissues when we examined the P2 promoter region. However, nearly half of the HCC cases displayed tumor-specific hypermethylation at both the P1 and P3 putative promoter sites. In fact, the number of CpG sites were more restricted in P2 than in the P1 and P3 regions. We also noticed only one *Bst*UI restriction site in P2 but two in the P1 region. P3 amplicons, which were devoid of *Bst*UI sites, were digested with the *Taq*I enzyme. Hypermethylation in the P3 alternative promoter region (43%) might inhibit *SIP1 *translation in a different context. The regulation of *SIP1 *translation by a NAT has been previously shown [27]. After the completion of EMT, a NAT is expressed and makes translation of *SIP1 *mRNA possible. This NAT expression was shown to be controlled by elements placed at the 5' site of the second exon, which corresponds to the P3 alternative promoter region [27]. Therefore, hypermethylation at the P3 site might inhibit the expression of the NAT, which in turn negatively affects *SIP1 *translation. It would be interesting to study the expression of the NAT in HCC.

Downregulation of SIP1 in HCC is also in accordance with the dual role of TGF-β in tumorigenesis. The tumor suppressor role of TGF-β in the premalignant stage was shown to switch to an EMT-inducing role in the later stages of cancers, leading to metastasis [47]. This former failsafe mechanism might partly explain higher levels of SIP1 expression in normal liver compared to HCCs. Despite our previous description that SIP1 is partly responsible for replicative senescence in liver cancer cells, its role in inducing apoptosis in distinct pathophysiolocal contexts should also be thoroughly investigated in HCC. Given the downregulation and possible tumor suppressor role of SIP1 in HCC, we also propose the assessment of this regulator as a prognostic factor for patients affected by this aggressive form of liver cancer.

## Conclusions

The data presented here clearly demonstrate that *SIP1 *is downregulated and undergoes epigenetic silencing in a considerable proportion of HCCs. Taken with our previous findings that SIP1 represses *hTERT *and mediates senescence arrest in HCC-derived cells, our results suggest that SIP1 is regulated in and a potential tumor suppressor gene of hepatocarcinogenesis.

## Competing interests

The authors declare that they have no competing interests.

## Authors' contributions

TA processed all the experiments except the IHC study and participated in the design of the study and in drafting the manuscript and data interpretation. EO performed the IHC study. TY participated in the IHC study and data interpretation and helped draft the manuscript. MCY designed and coordinated the study, participated in the data interpretation and in drafting the manuscript. All authors have read and approved the final manuscript.

## Pre-publication history

The pre-publication history for this paper can be accessed here:

http://www.biomedcentral.com/1471-2407/11/223/prepub

## Supplementary Material

Additional file 1 **Clinicopathological characteristics of liver samples used in quantitative RT-PCR**. Data about gender, age, diagnosis and tumor stage are given. A more detailed information on the samples can be found on the website below. http://www.origene.com/assets/documents/TissueScan/LVRT-01.xls Click here for file

Additional file 2 **Primers used in the study**. Name, sequence, Tm value and PCR product size are given. Click here for file

Additional file 3 **Sequence of the bisulfite-modified *SIP1 *putative promoter regions**. Arrows underlie the position of COBRA primers. Boxes show the cutting sites of the restriction enzymes. Click here for file

Additional file 4 **COBRA results of three SIP1 putative promoter regions in paired HCC samples**. **Figure S1**. *Bst*UI restriction analysis of the P1 region amplified by SIPM1dF/SIPM1iyR1 and SIPM1iF/SIPM1iyR1 semi-nested primer pairs. Fourteen out of 29 HCC samples (48%) are methylated. **Figure S2**. *Bst*UI restriction analysis of the P2 region amplified by SIPM2iyF1/SIPM2iR and SIPM2iyF1/SIPM2iyR2 semi-nested primer pairs. One out of 26 HCC samples (4%) is methylated. **Figure S3**. *Taq*I restriction analysis of the P3 region amplified by SIPM3dF/SIPM3dR and SIPM3iF/SIPM3iR nested primer pairs. Ten out of 23 HCC samples (43%) are methylated. Click here for file

## References

[B1] Ferlay J Bray F Pisani P Parkin DMJ eds GLOBOCAN 2000: Cancer Incidence, Mortality and Prevalence Worldwide 2001 Lyon: IARC Press Version 1.0

[B2] Parkin DM Bray FI Devesa SS Cancer burden in the year 2000. The global picture Eur J Cancer 2001 37 Suppl 8 S4 66 11602373 10.1016/s0959-8049(01)00267-2

[B3] Anthony PP MacSween R, Burt A, Portmann B, Ishak K, Scheuer P, Anthony P Tumors and tumor-like lesions of the liver and biliary tract: etiology, epidemiology and pathology Pathology of the Liver 2002 4 London, New York, Sydney, Toronto: Churchill Livingstone 711 775

[B4] Postigo AA Dean DC Differential expression and function of members of the zfh-1 family of zinc finger/homeodomain repressors Proc Natl Acad Sci USA 2000 97 12 6391 6396 10.1073/pnas.97.12.6391 10841546 PMC18613

[B5] Comijn J Berx G Vermassen P Verschueren K van Grunsven L Bruyneel E Mareel M Huylebroeck D van Roy F The two-handed E box binding zinc finger protein SIP1 downregulates E-cadherin and induces invasion Mol Cell 2001 7 6 1267 1278 10.1016/S1097-2765(01)00260-X 11430829

[B6] Van Grunsven LA Michiels C Van de Putte T Nelles L Wuytens G Verschueren K Huylebroeck D Interaction between Smad-interacting protein-1 and the corepressor C-terminal binding protein is dispensable for transcriptional repression of E-cadherin J Biol Chem 2003 278 28 26135 26145 10.1074/jbc.M300597200 12714599

[B7] Vandewalle C Comijn J De Craene B Vermassen P Bruyneel E Andersen H Tulchinsky E Van Roy F Berx G SIP1/ZEB2 induces EMT by repressing genes of different epithelial cell-cell junctions Nucleic Acids Res 2005 33 20 6566 6578 10.1093/nar/gki965 16314317 PMC1298926

[B8] Yoshimoto A Saigou Y Higashi Y Kondoh H Regulation of ocular lens development by Smad-interacting protein 1 involving Foxe3 activation Development 2005 132 20 4437 4448 10.1242/dev.02022 16162653

[B9] Verschueren K Remacle JE Collart C Kraft H Baker BS Tylzanowski P Nelles L Wuytens G Su MT Bodmer R Smith JC Huylebroeck D SIP1, a novel zinc finger/homeodomain repressor, interacts with Smad proteins and binds to 5'-CACCT sequences in candidate target genes J Biol Chem 1999 274 29 20489 20498 10.1074/jbc.274.29.20489 10400677

[B10] Thiery JP Chopin D Epithelial cell plasticity in development and tumor progression Cancer Metastasis Rev 1999 18 1 31 42 10.1023/A:1006256219004 10505544

[B11] Thiery JP Epithelial-mesenchymal transitions in development and pathologies Curr Opin Cell Biol 2003 15 6 740 746 10.1016/j.ceb.2003.10.006 14644200

[B12] Rosivatz E Becker I Specht K Fricke E Luber B Busch R Hofler H Becker KF Differential expression of the epithelial-mesenchymal transition regulators snail, SIP1, and twist in gastric cancer Am J Pathol 2002 161 5 1881 1891 10.1016/S0002-9440(10)64464-1 12414534 PMC1850763

[B13] Imamichi Y Konig A Gress T Menke A Collagen type I-induced Smad-interacting protein 1 expression downregulates E-cadherin in pancreatic cancer Oncogene 2007 26 16 2381 2385 10.1038/sj.onc.1210012 17043655

[B14] Isohata N Aoyagi K Mabuchi T Daiko H Fukaya M Ohta H Ogawa K Yoshida T Sasaki H Hedgehog and epithelial-mesenchymal transition signaling in normal and malignant epithelial cells of the esophagus Int J Cancer 2009 125 5 1212 1221 10.1002/ijc.24400 19431210

[B15] Elloul S Elstrand MB Nesland JM Trope CG Kvalheim G Goldberg I Reich R Davidson B Snail, Slug, and Smad-interacting protein 1 as novel parameters of disease aggressiveness in metastatic ovarian and breast carcinoma Cancer 2005 103 8 1631 1643 10.1002/cncr.20946 15742334

[B16] Sayan AE Griffiths TR Pal R Browne GJ Ruddick A Yagci T Edwards R Mayer NJ Qazi H Goyal S Fernandez S Straatman K Jones GD Bowman KJ Colquhoun A Mellon JK Kriajevska M Tulchinsky E SIP1 protein protects cells from DNA damage-induced apoptosis and has independent prognostic value in bladder cancer Proc Natl Acad Sci USA 2009 106 35 14884 14889 10.1073/pnas.0902042106 19706487 PMC2736415

[B17] Lin SY Elledge SJ Multiple tumor suppressor pathways negatively regulate telomerase Cell 2003 113 7 881 889 10.1016/S0092-8674(03)00430-6 12837246

[B18] Ozturk N Erdal E Mumcuoglu M Akcali KC Yalcin O Senturk S Arslan-Ergul A Gur B Yulug I Cetin-Atalay R Yakicier C Yagci T Tez M Ozturk M Reprogramming of replicative senescence in hepatocellular carcinoma-derived cells Proc Natl Acad Sci USA 2006 103 7 2178 2183 10.1073/pnas.0510877103 16461895 PMC1413736

[B19] Mejlvang J Kriajevska M Vandewalle C Chernova T Sayan AE Berx G Mellon JK Tulchinsky E Direct repression of cyclin D1 by SIP1 attenuates cell cycle progression in cells undergoing an epithelial mesenchymal transition Mol Biol Cell 2007 18 11 4615 4624 10.1091/mbc.E07-05-0406 17855508 PMC2043563

[B20] Miyoshi A Kitajima Y Sumi K Sato K Hagiwara A Koga Y Miyazaki K Snail and SIP1 increase cancer invasion by upregulating MMP family in hepatocellular carcinoma cells Br J Cancer 2004 90 6 1265 1273 10.1038/sj.bjc.6601685 15026811 PMC2409652

[B21] Oztas E Avci ME Ozcan A Sayan AE Tulchinsky E Yagci T Novel monoclonal antibodies detect Smad-interacting protein 1 (SIP1) in the cytoplasm of human cells from multiple tumor tissue arrays Exp Mol Pathol 2010 89 2 182 189 10.1016/j.yexmp.2010.05.010 20515682

[B22] Kojima T Takano K Yamamoto T Murata M Son S Imamura M Yamaguchi H Osanai M Chiba H Himi T Sawada N Transforming growth factor-beta induces epithelial to mesenchymal transition by down-regulation of claudin-1 expression and the fence function in adult rat hepatocytes Liver Int 2008 28 4 534 545 18031476 10.1111/j.1478-3231.2007.01631.x

[B23] Bracken CP Gregory PA Kolesnikoff N Bert AG Wang J Shannon MF Goodall GJ A double-negative feedback loop between ZEB1-SIP1 and the microRNA-200 family regulates epithelial-mesenchymal transition Cancer Res 2008 68 19 7846 7854 10.1158/0008-5472.CAN-08-1942 18829540

[B24] Christoffersen NR Silahtaroglu A Orom UA Kauppinen S Lund AH miR-200b mediates post-transcriptional repression of ZFHX1B RNA 2007 13 8 1172 1178 10.1261/rna.586807 17585049 PMC1924904

[B25] Park SM Gaur AB Lengyel E Peter ME The miR-200 family determines the epithelial phenotype of cancer cells by targeting the E-cadherin repressors ZEB1 and ZEB2 Genes Dev 2008 22 7 894 907 10.1101/gad.1640608 18381893 PMC2279201

[B26] Gregory PA Bert AG Paterson EL Barry SC Tsykin A Farshid G Vadas MA Khew-Goodall Y Goodall GJ The miR-200 family and miR-205 regulate epithelial to mesenchymal transition by targeting ZEB1 and SIP1 Nat Cell Biol 2008 10 5 593 601 10.1038/ncb1722 18376396

[B27] Beltran M Puig I Pena C Garcia JM Alvarez AB Pena R Bonilla F de Herreros AG A natural antisense transcript regulates Zeb2/Sip1 gene expression during Snail1-induced epithelial-mesenchymal transition Genes Dev 2008 22 6 756 769 10.1101/gad.455708 18347095 PMC2275429

[B28] Rodenhiser DI Andrews J Kennette W Sadikovic B Mendlowitz A Tuck AB Chambers AF Epigenetic mapping and functional analysis in a breast cancer metastasis model using whole-genome promoter tiling microarrays Breast Cancer Res 2008 10 4 R62 10.1186/bcr2121 18638373 PMC2575535

[B29] Li A Omura N Hong SM Vincent A Walter K Griffith M Borges M Goggins M Pancreatic cancers epigenetically silence SIP1 and hypomethylate and overexpress miR-200a/200b in association with elevated circulating miR-200a and miR-200b levels Cancer Res 2010 70 13 5226 5237 10.1158/0008-5472.CAN-09-4227 20551052 PMC3130565

[B30] Ozturk M p53 mutation in hepatocellular carcinoma after aflatoxin exposure Lancet 1991 338 8779 1356 9 10.1016/0140-6736(91)92236-U 1682737

[B31] Avci ME Konu O Yagci T Quantification of SLIT-ROBO transcripts in hepatocellular carcinoma reveals two groups of genes with coordinate expression BMC Cancer 2008 8 392 10.1186/1471-2407-8-392 19114000 PMC2632672

[B32] Fu LY Jia HL Dong QZ Wu JC Zhao Y Zhou HJ Ren N Ye QH Qin LX Suitable reference genes for real-time PCR in human HBV-related hepatocellular carcinoma with different clinical prognoses BMC Cancer 2009 9 49 10.1186/1471-2407-9-49 19200351 PMC2644316

[B33] Gur-Dedeoglu B Konu O Bozkurt B Ergul G Seckin S Yulug IG Identification of endogenous reference genes for qRT-PCR analysis in normal matched breast tumor tissues Oncol Res 2009 17 8 353 365 10.3727/096504009788428460 19544972

[B34] Pfaffl MW A new mathematical model for relative quantification in real-time RT-PCR Nucleic Acids Res 2001 29 9 e45 10.1093/nar/29.9.e45 11328886 PMC55695

[B35] Chen CL Hsieh FC Lin J Systemic evaluation of total Stat3 and Stat3 tyrosine phosphorylation in normal human tissues Exp Mol Pathol 2006 80 295 305 10.1016/j.yexmp.2005.11.003 16427042

[B36] Datta J Kutay H Nasser MW Nuovo GJ Wang B Majumder S Liu CG Volinia S Croce CM Schmittgen TD Ghoshal K Jacob ST Methylation mediated silencing of MicroRNA-1 gene and its role in hepatocellular carcinogenesis Cancer Res 2008 68 13 5049 5058 10.1158/0008-5472.CAN-07-6655 18593903 PMC2562630

[B37] Huang J Zhang YL Teng XM Lin Y Zheng DL Yang PY Han ZG Down-regulation of SFRP1 as a putative tumor suppressor gene can contribute to human hepatocellular carcinoma BMC Cancer 2007 7 126 10.1186/1471-2407-7-126 17626620 PMC1940018

[B38] Mazieres J Tovar D He B Nieto-Acosta J Marty-Detraves C Clanet C Pradines A Jablons D Favre G Epigenetic regulation of RhoB loss of expression in lung cancer BMC Cancer 2007 7 220 10.1186/1471-2407-7-220 18047684 PMC2222678

[B39] Knudsen S Promoter2.0: for the recognition of PolII promoter sequences Bioinformatics 1999 15 5 356 361 10.1093/bioinformatics/15.5.356 10366655

[B40] Li LC Dahiya R MethPrimer: designing primers for methylation PCRs Bioinformatics 2002 18 11 1427 1431 10.1093/bioinformatics/18.11.1427 12424112

[B41] Xiong Z Laird PW COBRA: a sensitive and quantitative DNA methylation assay Nucleic Acids Res 1997 25 12 2532 2534 10.1093/nar/25.12.2532 9171110 PMC146738

[B42] Nelles L Van de Putte T van Grunsven L Huylebroeck D Verschueren K Organization of the mouse Zfhx1b gene encoding the two-handed zinc finger repressor Smad-interacting protein-1 Genomics 2003 82 4 460 469 10.1016/S0888-7543(03)00169-1 13679026

[B43] Julien S Puig I Caretti E Bonaventure J Nelles L van Roy F Dargemont C Garcia Herreros A Bellacosa A Larue L Activation of NF-κB by Akt upregulates Snail expression and induces epithelium mesenchyme transition Oncogene 2007 26 7445 7456 10.1038/sj.onc.1210546 17563753

[B44] Wurmbach E Chen YB Khitrov G Zhang W Roayaie S Schwartz M Fiel I Thung S Mazzaferro V Bruix J Bottinger E Friedman S Waxman S Llovet JM Genome-wide molecular profiles of HCV-induced dysplasia and hepatocellular carcinoma Hepatology 2007 45 4 938 947 10.1002/hep.21622 17393520

[B45] Esteller M Cancer epigenomics: DNA methylomes and histone-modification maps Nat Rev Genet 2007 8 4 286 298 10.1038/nrg2005 17339880

[B46] Ladeiro Y Couchy G Balabaud C Bioulac-Sage P Pelletier L Rebouissou S Zucman-Rossi J MicroRNA profiling in hepatocellular tumors is associated with clinical features and oncogene/tumor suppressor gene mutations Hepatology 2008 47 6 1955 1963 10.1002/hep.22256 18433021

[B47] Roberts AB Wakefield LM The two faces of transforming growth factor beta in carcinogenesis Proc Natl Acad Sci USA 2003 100 15 8621 8623 10.1073/pnas.1633291100 12861075 PMC166359

