# Supplementary material for: SIP1 is downregulated in hepatocellular carcinoma by promoter hypermethylation
Source: BMC Cancer. 2011 Jun 6;11:223. doi: 10.1186/1471-2407-11-223 (PMC3118200; doi:10.1186/1471-2407-11-223)
Supplement: Additional file 2 — Primers used in the study. Name, sequence, Tm value and PCR product size are given. [file 1471-2407-11-223-S2.PDF]

**Additional file 2 :** Primers used in the study

| Primer | Sequence | Tm (C°) | Product Size (bp) |
|---|---|---|---|
| Sip1F | caatgatggcactctgtgga | 60.69 | 416 |
| Sip1R | tgccaatcaaagcaatatcgt | 60.47 | |
| Sip2F | ctcaaaccttttggcctgtct | 60.65 | 538 |
| Sip2R | tgccagttgagtttcctcag | 59.01 | |
| Sip3F | tggttcctacagttcgcaca | 60.30 | 569 |
| Sip3R | cgacaggcggaatattagga | 60.05 | |
| Sip4F | tgcagcctcatgaaaacatagt | 59.77 | 439 |
| Sip4R | agcctgagaggaggatcaca | 59.94 | |
| SE2F | tccctcatacggtcaggagttat | 61.07 | 509 |
| SE2R | cctcggttccttttcccttt | 61.64 | |
| SE3F | caattaggggtggctgatgt | 59.81 | 415 |
| SE3R | tatttggttgtgggcgatct | 60.33 | |
| SE4F | ttgtcactagaactgccacca | 59.35 | 422 |
| SE4R | tccttccctgcctcactaaa | 59.81 | |
| SE5F | atcggtcatcttcaacacttctt | 59.16 | 422 |
| SE5R | aggtaaacacccaggcatgt | 59.33 | |
| SE81F | ggtaccccattgtgttccttt | 59.97 | 729 |
| SE81R | cgacaggcggaatattagga | 60.05 | |
| SE82F | tctcaacctgaggaacaagga | 59.83 | 543 |
| SE82R | tttcagcagttcatcggagtt | 59.87 | |
| SE83F | tcaccatctatagcagaactcca | 58.87 | 722 |
| SE83R | aatcaaaataattgccacctctt | 58.17 | |
| SE9F | tgaagttgtttggtgtgagca | 60.33 | 395 |
| SE9R | agtcctactgagctcggcaa | 60.16 | |
| SE10F | agtggaaagagacttcatgcaaa | 60.29 | 705 |
| SE10R | cagtgttttcaagcaggtaacaata | 59.66 | |
| SIPM1dF | agaaaggagaacgtaggaaatgtaat | 59.13 | 369 |
| SIPM1iyR1 | aaaaataataccgaaaactaattaccata | 57.6 | |
| SIPM1iF | aaggagggttagagggaggaaaagt | 63.87 | 296 |
| SIPM1iyR1 | aaaaataataccgaaaactaattaccata | 57.6 | |
| SIPM2iyF1 | gaggaggaagggagggaggt | 63.13 | 380 |
| SIPM2iR | aacaactcccgaaacaaactataca | 60.20 | |

| Continued | | | |
|---|---|---|---|
| **Primer** | **Sequence** | **Tm (C°)** | **Product Size (bp)** |
| SIPM2iyF1 | gaggaggaagggagggaggt | 63.13 | 281 |
| SIPM2iyR2 | actccaaaaaacacaaacctaaaa | 58.3 | |
| SIPM3dF | ttagtaaatgtgtggaaattgatatt | 55.43 | 800 |
| SIPM3dR | cttttcgttctcatcttttctcat | 58.16 | |
| SIPM3iF | cggtagagaaagggttaatggtt | 59.79 | 631 |
| SIPM3iR | aaacaacccctaaataaaacacact | 58.49 | |
| SIP1-RTF | tgtagatggtccagaagaaatgaa | 60.00 | 132 |
| SIP1-RTR | ttggcaaagtattcctcaaaatct | 60.34 | |
| GAPDH-RTF | agtcaacggatttggtcgtatt | 59.8 | 611 |
| GAPDH-RTR | gtagaggcagggatgatgttct | 59.6 | |
| TBP-RTF* | tgcacaggagccaagagtgaa | 64.4 | 132 |
| TBP-RTR* | cacatcacagctccccacca | 65.6 | |

(*) These primer sequences were obtained from Gur-Dedeoglu B. *et al.* (2009)
