# Supplementary material for: SIP1 is downregulated in hepatocellular carcinoma by promoter hypermethylation
Source: BMC Cancer. 2011 Jun 6;11:223. doi: 10.1186/1471-2407-11-223 (PMC3118200; doi:10.1186/1471-2407-11-223)
Supplement: Additional file 3 — Sequence of the bisulfite-modified SIP1 putative promoter regions. Arrows underlie the position of COBRA primers. Boxes show the cutting sites of the restriction enzymes. [file 1471-2407-11-223-S3.PDF]

Additional file 3 : Sequence of the bisulfite-modified SIP1 putative promoter regions. Arrows underlie the position of COBRA primers. Boxes show the cutting sites of the restriction enzymes.

P1 region analyzed by *Bst*UI

tagat**agaaaggagaacgtaggaaatgtaat**tgggaaagaattttaagaaaataaggata
SIPM1dF
aagggtagggagggttga**aaggagggttagagggaggaaaagt**aggagggagatttttgg
SIPM1iF
tttttaattttttggtgaggttgttggcgtt**cgcg**tttttaacgttttagttttttaagt
tgaggcgaaagttatttttcgggtttgcgtcgtatttaatttattttaagttttttggt
gattttatagggttttttttcgttttttttttcgtttttcgttttttaagttttagtga
gattgatagt**cgcg**tggaggaggtttttttaggtatagttttttt**tatggtaattagttt**
tcggtattatttttcggtcgcggttgcgttcgttcggagcgcggttcgcggatttcgggg
SIPM1iyR1

P2 region analyzed by *Bst*UI

tggaggggggagaagggagggaggggggagggaggcgaaggcgaaaggaggga**gagga**
SIPM2iyF1
ggaagggagggaggtggaattttattttttttttattaaagcgtttgcggagattttaaggt
ataatttattttagatttttttttagagagaaatttggcgattacgttttatatgatgt
ttacgtttagggcgttttaattatttttttttataaagataggtgg**cgcg**tgttttaggg
tttttcgtttttttttttatagaaaagaaaaagaaaaaaatgttattagaagaggcgtaat
acgttagttcgt**ttttaggtttgtgtttttggagt**ggtcgaaagagattagttttaatt
SIPM2iyR2
tgttttgtaggaataacggtttgtttttcgatattttggcgaggtttt**tgtatagttt**
gtttcgggagttgttttttcgttttatttttttttttttttatatttcgcggttttttta
SIPM2iR

P3 region analyzed by *Taq*I

tttttttatttttt**ttagtaaatgtgtggaaattgatatt**tattttttaggttttttgta
SIPM3dF
aagtttttagtataggagataataggtgtggggcgtggggattgagtgtgtt**cggtagag**
SIPM3iF
aaagggttaatggtttttgtgttatagtttgtatttgttatattttttatagttttttgt
ttaaattaattt**tcga**ggtatagtatttttttaattttta**tcga**gtttttaggatttttt
tttttttttgtttgttt**tcga**ggttttttttttttaagttttgatagttttaattgaaaa
aatgtagcgtttttt**tcga**tttttaggtataaaagtt**tcga**agttagaaaggatacgatt
atttagtagagtttaaaagtattgtcgtagtggttggattttttttttttttttttaagg
gaaaaaaaaatttttattggaattatttaaggaattagtgtggggggagtatgtgtgtga
gtgaggtggggggaaatatttaagtaaatattgttaaagtggcgaggggggggtggggtaa
aggatagtgtttaaagaggtttataattatatttattattgcggcgtaaaacggaaatt
ttaatttgggggcgagggtggggggaggaagagatagtgtt**tcga**taaattttaaaaggg
gaggggagggggcgttgggcgagtgggttttttataattattattattaatttgt**tcgag**
tgtgttttatttaggggttgtttcgcgttgtttgtttgtttgttttttttgggaattggg
SIPM3iR
gggtgggggg**atgagaaaagatgagaacgaaaag**aaaatttcgttttatttaagtttt
SIPM3dR
