# Supplementary material for: SIP1 is downregulated in hepatocellular carcinoma by promoter hypermethylation
Source: BMC Cancer. 2011 Jun 6;11:223. doi: 10.1186/1471-2407-11-223 (PMC3118200; doi:10.1186/1471-2407-11-223)
Supplement: Additional file 4 — COBRA results of three SIP1 putative promoter regions in paired HCC samples. Figure S1. BstUI restriction analysis of the P1 region amplified by SIPM1dF/SIPM1iyR1 and SIPM1iF/SIPM1iyR1 semi-nested primer pairs. Fourteen out of 29 HCC samples (48%) are methylated. Figure S2. BstUI restriction analysis of the P2 region amplified by SIPM2iyF1/SIPM2iR and SIPM2iyF1/SIPM2iyR2 semi-nested primer pairs. One out of 26 HCC samples (4%) is methylated. Figure S3. TaqI restriction analysis of the P3 region amplified by SIPM3dF/SIPM3dR and SIPM3iF/SIPM3iR nested primer pairs. Ten out of 23 HCC samples (43%) are methylated. [file 1471-2407-11-223-S4.PDF]

Additional file 4 : COBRA results of three SIP1 putative promoter regions in paired HCC samples.

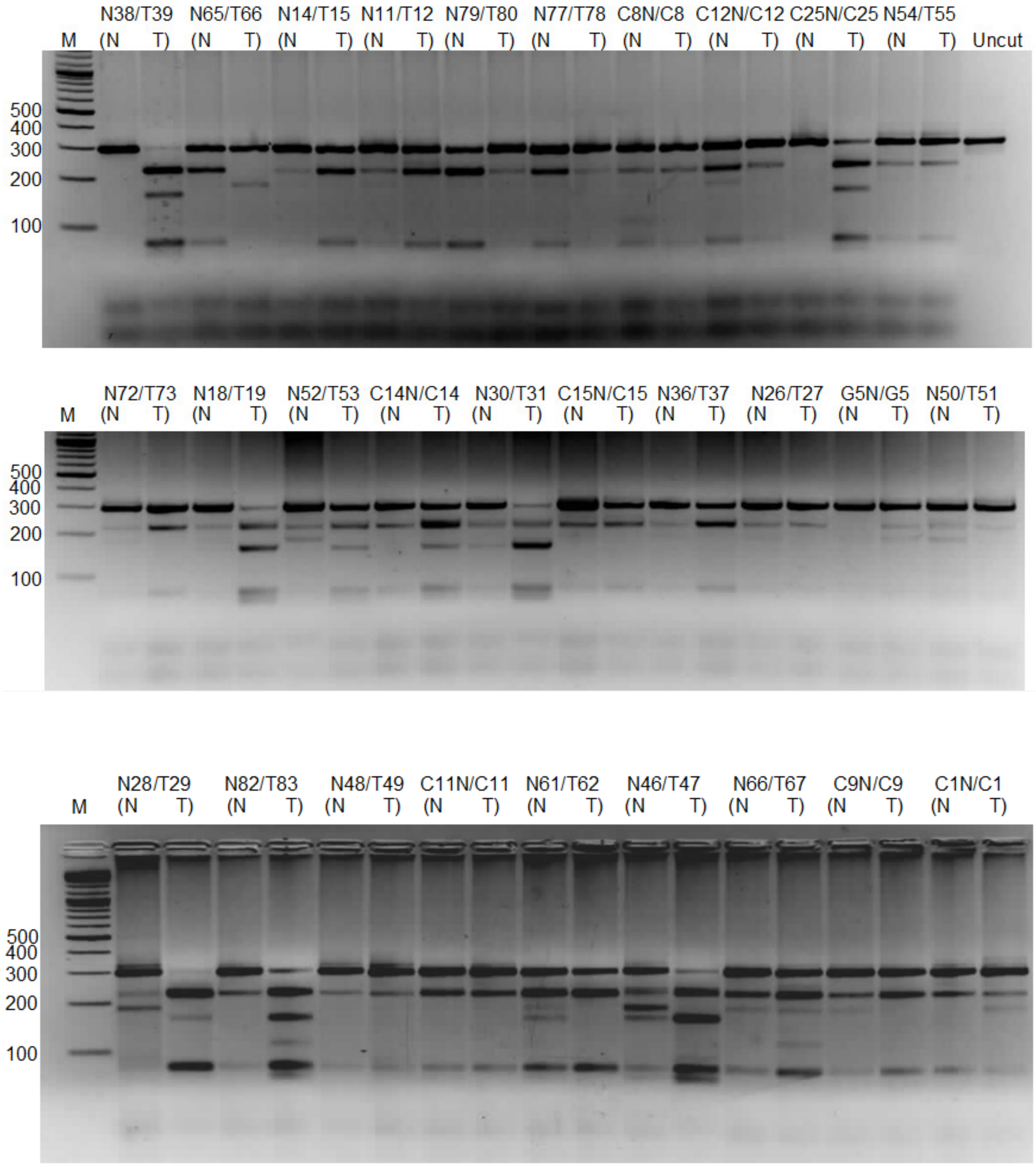

Figure S1.

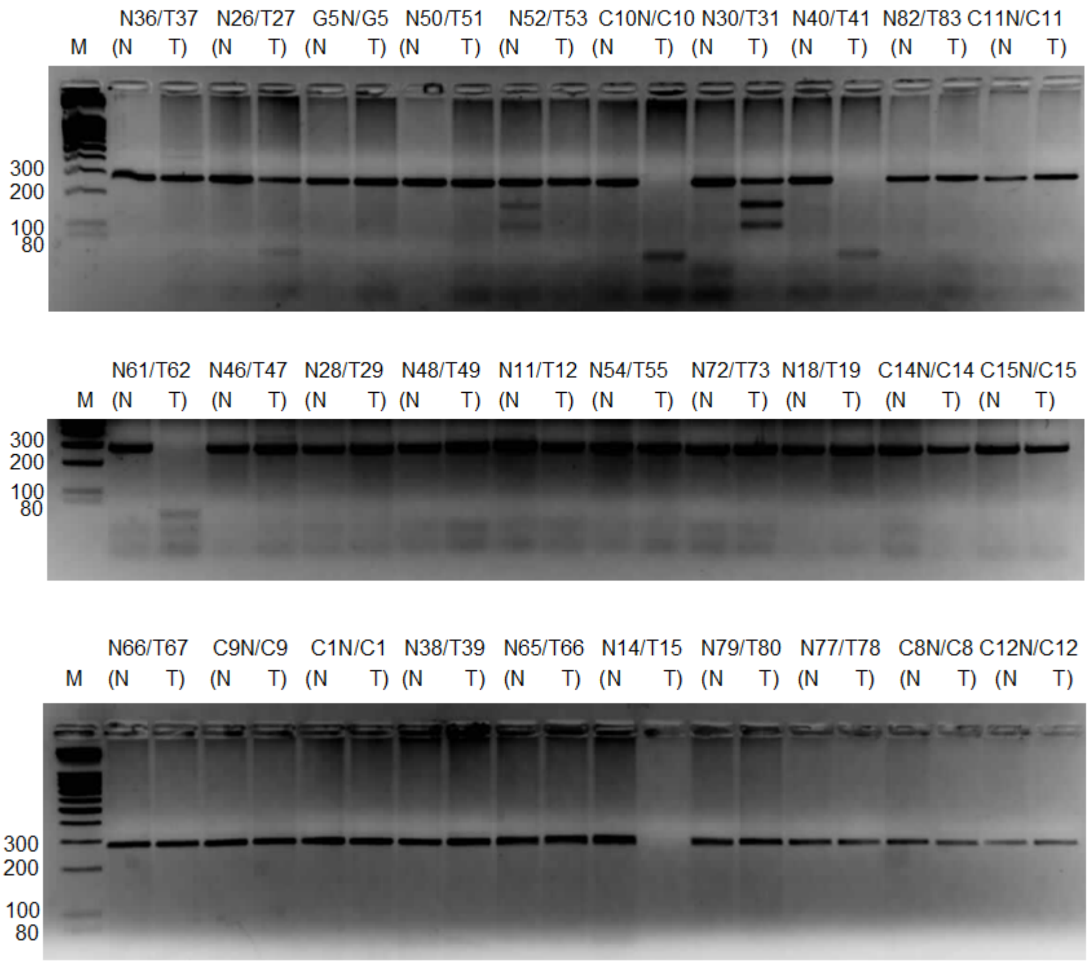
N36/T37 N26/T27 G5N/G5 N50/T51 N52/T53 C10N/C10 N30/T31 N40/T41 N82/T83 C11N/C11
M (N T) (N T) (N T) (N T) (N T) (N T) (N T) (N T) (N T) (N T)
300
200
100
80
N61/T62 N46/T47 N28/T29 N48/T49 N11/T12 N54/T55 N72/T73 N18/T19 C14N/C14 C15N/C15
M (N T) (N T) (N T) (N T) (N T) (N T) (N T) (N T) (N T) (N T)
300
200
100
80
N66/T67 C9N/C9 C1N/C1 N38/T39 N65/T66 N14/T15 N79/T80 N77/T78 C8N/C8 C12N/C12
M (N T) (N T) (N T) (N T) (N T) (N T) (N T) (N T) (N T) (N T)
300
200
100
80

Figure S2.

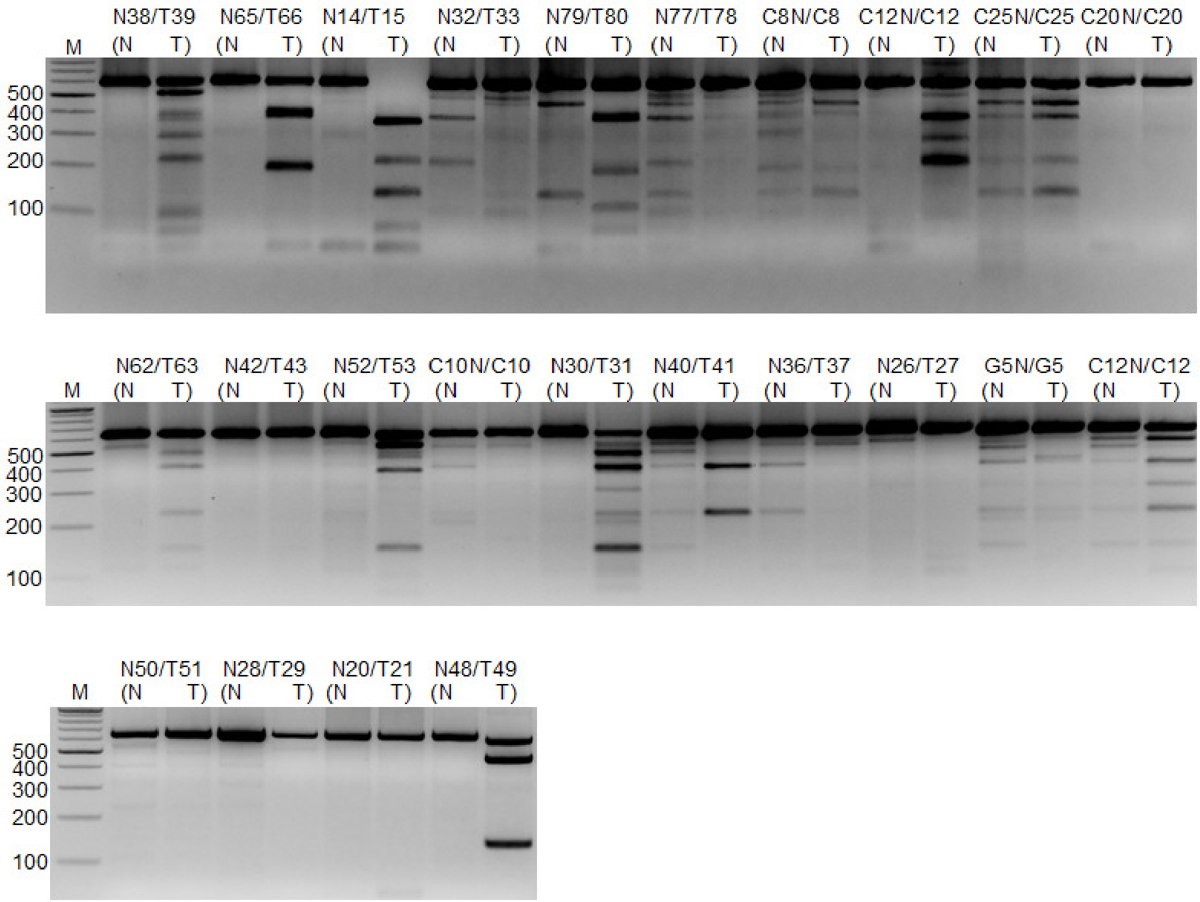
M
N38/T39
N65/T66
N14/T15
N32/T33
N79/T80
N77/T78
C8N/C8
C12N/C12
C25N/C25
C20N/C20
500
400
300
200
100
N62/T63
N42/T43
N52/T53
C10N/C10
N30/T31
N40/T41
N36/T37
N26/T27
G5N/G5
C12N/C12
N50/T51
N28/T29
N20/T21
N48/T49

Figure S3.
